# Elevated Urinary T Helper 1 Chemokine Levels in Newly Diagnosed Hypertensive Obese Children

**DOI:** 10.4274/jcrpe.1917

**Published:** 2015-08-31

**Authors:** Duygu Övünç Hacıhamdioğlu, Cengiz Zeybek, Faysal Gök, Aysel Pekel, Uğur Muşabak

**Affiliations:** 1 Gülhane Military Medical Academy, Haydarpaşa Training Hospital, Clinic of Child Health and Diseases, İstanbul, Turkey; 2 Gülhane Military Medical Academy Hospital, Department of Child Health and Diseases, Ankara, Turkey; 3 Gülhane Military Medical Academy Hospital, Department of Immunology, Ankara, Turkey

**Keywords:** hypertension, obesity, children, urine MIG, urine IP10

## Abstract

**Objective:**

Increasing evidence suggests that T helper (Th) cells play a significant role in the pathogenesis of hypertension. The aim of this study was to evaluate the effect of obesity and anti-hypertensive treatment on urinary Th1 chemokines.

**Methods:**

The study groups consisted of three types of patients: hypertensive obese, healthy, and non-hypertensive obese. Pre-treatment and post-treatment samples of the hypertensive obese group and one sample from the other two groups were evaluated for urinary chemokine: regulated on activation, normal T cell expressed and secreted (RANTES), interferon-gamma-inducible protein 10 (IP10), and monokine induced by interferon-gamma (MIG). In the hypertensive obese group, urine microalbumin: creatinine ratio was examined before and after treatment. We recommended lifestyle changes to all patients. Captopril was started in those who could not be controlled with lifestyle changes and those who had stage 2 hypertension.

**Results:**

Twenty-four hypertensive obese (mean age 13.1), 27 healthy (mean age 11.2) and 22 non-hypertensive obese (mean age 11.5) children were investigated. The pre-treatment urine albumin: creatinine ratio was positively correlated with pre-treatment MIG levels (r=0.41, p<0.05). RANTES was significantly higher in the pre-treatment hypertensive and non-hypertensive obese group than in the controls. The urinary IP10 and MIG levels were higher in the pre-treatment hypertensive obese group than in the non-hypertensive obese. Comparison of the pre- and post-treatment values indicated significant decreases in RANTES, IP10, and MIG levels in the hypertensive obese group (p<0.05).

**Conclusion:**

Th1 cells could be activated in obese hypertensive children before the onset of clinical indicators of target organ damage. Urinary RANTES seemed to be affected by both hypertension and obesity, and urinary IP10 and MIG seemed to be affected predominantly by hypertension.

## INTRODUCTION

Obesity is a common health problem worldwide; major consequences of obesity include an increased prevalence of hypertension and a cascade of associated renal disorders. The mechanisms by which obesity cause renal injury other than hypertension remain unclear, although the importance of inflammation has been suggested (1).

Adipose tissue is a source of inflammatory cytokines such as C-reactive protein (CRP), interleukin (IL)-6, and tumor necrosis factor-alpha (TNF-α) (2). Chronic inflammation plays a central role in the pathophysiology of hypertension and associated end-organ damage, including renal injury (3,4,5,6).

Increasing evidence suggests that T cells play a significant role in the pathogenesis of hypertension (7). One of the main functions of T helper 1 (Th1) cells is the secretion of interferon-gamma (IFN-γ), which promotes the expression of cytokines, adhesion molecules, and chemokines (8,9). Low-molecular-weight proteins of the cytokine family called chemokines, which have the ability to stimulate and control leukocyte migration, participate in an inflammatory reaction involving the vascular wall in hypertension (10). Th1-associated chemokines, including regulated on activation, normal T cell expressed and secreted (RANTES), interferon-inducible protein 10 (IP10), and monokine induced by IFN-γ (MIG), have been detected in adults with coronary artery disease, aortic aneurysms, or essential hypertension with microalbuminuria (11,12,13,14,15).

In recent studies, urine has emerged as a potentially more suitable reservoir than blood for identifying biomarkers. In contrast to blood, preanalytical handling is simple, and urine is stable. Approximately 70% of urinary proteins originate from the kidneys and urinary tract in healthy individuals, and the percentage might be even higher in individuals with kidney disease (16).

No studies have examined the association between urinary chemokines and hypertension in pediatric patients. Therefore, in this study, we aimed to evaluate the effect of obesity and anti-hypertensive treatment on the urinary Th1 chemokines IP10, MIG, and RANTES in hypertensive obese children.

## METHODS

### Patients

This prospective study examined three groups of patients (hypertensive obese, non-hypertensive obese, and control) from the Pediatric Nephrology Clinic at our hospital. All patients provided written informed consent, and our institutional ethics committee approved the study protocol.

All hypertensive subjects had a history of in-office systolic or diastolic blood pressure (BP) measurements ≥95th percentile on at least three occasions (17). Hypertension was confirmed by 24-hour ambulatory BP monitoring (ABPM), in which hypertension was defined as a mean daytime or nighttime BP ≥95th percentile for pediatric ambulatory norms (18). Obesity was defined as a body mass index (BMI) ≥95th percentile for age and sex. Non-obesity was defined as BMI <85th percentile for age and sex. Normotension was defined as a systolic or diastolic BP <90th percentile according to age, sex, and height, and confirmed by ABPM. Dyslipidemia was defined as total cholesterol >200 mg/dL, low-density lipoprotein (LDL) cholesterol >130 mg/dL, or triglyceride (TG) level >130 mg/dL in children older than 10 years. Pharmacotherapy is initiated in older children with an elevated LDL cholesterol or TG level when dietary measures fail to achieve target levels after 6 months (19). Insulin resistance (IR) was defined according to the HOMA-IR (homeostatic model assessment of IR) (20).

The exclusion criteria were the use of cigarettes, dyslipidemia requiring pharmacological treatment, and the presence of any other disease, including white coat hypertension, neuropsychological abnormalities, obstructive sleep apnea, chronic diseases, left ventricular hypertrophy, retinopathy, secondary hypertension (e.g., primary hyperaldosteronism or glucocorticoid remediable hypertension), IR, hirsutism, menstrual irregularities (for females), episodes of complete or partial upper airway obstruction during sleep, and elevated liver transaminases (alanine aminotransferase, aspartate aminotransferase, alkaline phosphatase, and gamma glutamyl transpeptidase).

The inclusion criteria for the non-hypertensive obese and control groups were as follows: no chronic disease, no family history of chronic disease such as stroke, diabetes, or dyslipidemia, no use of any medications during the study period or in the preceding 6 weeks, and no existing or previous infections in the 6 weeks before the study according to the patient’s clinical history and a physical examination.

Newly diagnosed, untreated hypertensive obese children were assigned to the hypertensive obese group. The healthy control group consisted of normotensive and non-obese subjects. Normotensive obese children served as the non-hypertensive obese group.

We recommended lifestyle changes to all patients, including dietary salt (4.25-5.0 g/m2) and fat restrictions, a diet consisting mainly of fruits and vegetables, 50% reduction in dairy products, 30-60 minutes of aerobic exercise 3 days per week, no smoking, minimal exposure to caffeine and energy drinks, and restricted television and computer time (no more than 2 hours per day). The patients receive dietary counseling regarding dietary changes. Anti-hypertensive treatment was started in those who could not be controlled by lifestyle changes after 2 months and those with stage 2 hypertension. Our patients were given captopril in a dose of 12.5 mg every 12 hours, titrated as needed, with a maximum daily dose of 100 mg.

In all groups, ABPM and urinary chemokine measurements were obtained. Blood chemistry and urine examinations were done to determine the microalbumin: creatinine ratio in the hypertensive obese group during the pre- and post-treatment periods. Post-treatment samples from the hypertensive obese group were collected after BP control had been established for at least 2 months. The samples for the non-hypertensive obese group were collected before the diet and exercise recommendations for weight loss.

### Ambulatory Blood Pressure Monitoring

ABPM was performed using Mobil-O-Graph NG monitors (IEM, Stolberg, Germany). Monitors were programmed to obtain BP readings every 15 minutes from 07:00 to 22:00, and every 30 minutes from 22:00 to 07:00. Wake and sleep periods for ABPM were determined from the patients’ self-reports. BP was analyzed using Mobil-O-Graph software (ABP Hypertension Management System CS ver.; IEM).

The number of measurements, mean arterial pressure, pulse pressure, systolic and diastolic load, and dipping were evaluated by ABPM. BP load in children was defined as the percentage over the normal BP measurements according to the time of the age and height measurements (21). Patients with a >10% decrease in the night/sleep time mean BP compared with the daytime/awake mean BP were considered dipper patients; all others were considered non-dipper patients (22). The difference between the systolic and diastolic pressures, which generates the pulse, was designated as the pulse pressure.

### Biochemical Analyses

The albumin: creatinine ratio was evaluated using the first morning urine. Urinary albumin was measured by immunonephelometry (Siemens Healthcare Diagnostics Products GmbH, Marburg, Germany), with a threshold of 42-52 g/L and intra- and inter-assay coefficients of variation of <3.5% and 1.8%, respectively. Serum urea, creatinine, uric acid, cholesterol, and TGs were measured colorimetrically (AU 2700 autoanalyzer; Olympus, Hamburg, Germany). The urinary N-acetyl-β-glucosaminidase level was measured colorimetrically in spot urine at a wavelength of 405 nm (Cintra 303 spectrophotometer; GBC Scientific Equipment, Castle Hill, New South Wales, Australia). The data were normalized based on creatinine, and the results are presented in U/mmol-creatinine (reference range: 0.21-2.5 U/mmol-creatinine). Blood samples were collected from the subjects in the hypertensive obese group before and after treatment.

### Urinary Chemokine Detection

Pre- and post-treatment samples from the hypertensive obese group and only one sample from the non-hypertensive obese and control groups were evaluated for urinary chemokines. Fresh first morning urine after initial voiding was collected in a standardized manner, centrifuged immediately at 2000×g, 4 °C, and aliquots of the supernatant were collected in appropriate sterile Eppendorf tubes and frozen immediately at -80 °C until evaluation of the immune mediators. RANTES, IP10, and MIG levels were examined by flow cytometry. To detect chemokines in the samples, a Cytometric Bead Array kit (BD Biosciences, San Jose, CA, USA) was used. Samples prepared at room temperature were evaluated on the same day using Fcap Array V3.0 software on a Model FACSCanto flow cytometer (BD Biosciences).

### Statistical Analysis

All statistical analyses were performed using SPSS for Windows 15.0 (SPSS, Chicago, IL, USA). The means in more than two groups were compared with Kruskal-Wallis and one-way ANOVA test. When overall p-value was significant, then pairwise comparisons were done with Mann-Whitney U-test and post-hoc Bonferroni test. The means in two groups were compared with Mann-Whitney and Wilcoxon signed-rank tests. Non-normally distributed parameters are expressed as medians (25th and 75th percentiles), while normally distributed parameters are expressed as means ± standard deviation. In all analyses, p<0.05 was taken to indicate statistical significance. The Bonferroni correction was applied for comparisons of subgroups (p<0.05/number of comparisons). A non-parametric correlation analysis (Spearman’s rank correlation) was also performed.

## RESULTS

### Demographics, Ambulatory Blood Pressure Monitoring, and Biochemical Features

The demographic characteristics of the patients are shown in Table 1. When we compared the three groups for BMI, we found differences between groups (p=0.004). In pairwise comparisons, there was no difference in BMI between the hypertensive obese group and the non-hypertensive obese group (p=0.305).

The comparison of the ABPM parameters between pre-treatment hypertensive obese and other groups are shown in Table 2. Compared to the non-hypertensive obese and control groups, daytime diastolic load was higher in the non-hypertensive obese group than in the controls (p=0.024). After treatment, there were no differences in ABPM parameters between the hypertensive obese group and the control group (Table 3). The mean time between the pre- and post-treatment samples was 5 (range 2-9) months. Nine subjects (37.5%) in the hypertensive obese group received captopril as medication. No significant decrease in mean BMI was observed in the hypertensive obese group (29.9 kg/m2) after treatment (p>0.05).

Table 4 shows the pre-treatment and post-treatment biochemical parameters of the hypertensive obese group. Post-treatment levels of uric acid, total cholesterol, LDL, TG, urine NAG, and urine microalbumin: creatinine ratio were all lower than pre-treatment levels.

### Urinary Chemokine Results

Table 5 shows the urinary chemokine results and a comparison of the pre-treatment hypertensive obese and the other groups. The comparison of the urinary chemokine between post-treatment hypertensive obese and the other groups are shown in Table 6. Urine chemokine results were non-normally distributed parameters by Kolmogorov-Smirnov test. Therefore, the means between the groups were compared with Kruskal-Wallis test; the results are expressed as medians (25th and 75th percentiles). The pairwise comparisons were done using the Mann-Whitney test. The comparison of hypertensive obese regarding pre-treatment and post-treatment values was performed with Wilcoxon signed-rank test.

RANTES was not different between the pre-treatment hypertensive obese and non-hypertensive obese groups, but it was higher in the pre-treatment hypertensive obese and non-hypertensive obese groups than the controls (p=0.002 and p=0.001, respectively). After the treatment, urine RANTES was reduced in the hypertensive obese group (p=0.005). While IP10 was higher in the pre-treatment hypertensive group compared to non-hypertensive obese and control subjects (p=0.000 and p=0.001, respectively), there was no difference between non-hypertensive obese and control groups. After the treatment, urine IP10 was reduced in the hypertensive obese group (p=0.000).

The pre-treatment MIG level was higher in the hypertensive obese group than the non-hypertensive obese group (p=0.006). After the treatment, urine MIG was reduced in the hypertensive obese group (p=0.010).

In a linear correlation analysis, the pre-treatment urine albumin: creatinine ratio in the hypertensive obese group was correlated positively with the pre-treatment MIG level (r=0.41, p=0.046) and daytime diastolic load (r=0.48, p=0.018). Therve were no correlations between ABPM, biochemical, or urinary chemokine parameters in the pre-treatment hypertensive obese group. The hypertensive obese group was divided into two subgroups, consisting of patients treated with captopril (n=9) and those controlled using lifestyle changes only (n=15). There were no significant differences between the two subgroups in urinary chemokines or microalbuminuria in the pre- and post-treatment periods.

## DISCUSSION

Obesity has become an important medical problem in children, and primary hypertension in children has become increasingly common in association with obesity. However, the mechanisms underlying obesity-associated hypertension are unclear (1,2,3). The results of this study suggest that Th1-mediated inflammation is active in children with obesity and hypertension, and that antihypertensive therapy involving captopril and/or lifestyle changes can reverse this condition.

In this study, serum TGs, cholesterol, and blood pressure were decreased by lifestyle changes alone in 15 patients. A recent systematic review and meta-analysis of randomized clinical trials assessing the effects of physical activity interventions on the prevention of cardiovascular risk factors in childhood indicated that physical activity interventions were not associated with reductions in BMI. However, there were associations between the interventions and reductions in systolic BP, diastolic BP, and TGs (23). Consequently, weight loss, aerobic exercise, and diet might be capable of changing the hypertensive state.

The chronic inflammatory state associated with obesity contributes to the pathogenesis of hypertension. In our study, urinary RANTES was reduced after treatment in the hypertensive obese group, but it was higher in the non-hypertensive obese group than in the controls. Elevated RANTES secretion has been reported in the adipose tissue of obese patients (24,25). Furthermore, it was reported that while RANTES mRNA and protein expression levels were elevated in the kidneys of hypertensive rats, the level of Na+-ATPase activity was increased. In addition, suppression of inflammation was associated with the normalization of Na+-ATPase activity, reduced renal injury, and the amelioration of hypertension (26). Th1 chemokines might play an important role in the hypertensive state. Further research focusing on Th1 chemokines may provide a better understanding of the pathophysiology of obesity-associated hypertension.

Urinary IP10 and MIG were involved more in the hypertensive obese than in the non-hypertensive obese subjects. Furthermore, the pre-treatment MIG level was correlated with the urinary albumin: creatinine ratio. Urinary MIG and IP10 were shown to be involved in the earliest aspect of inflammatory conditions, including acute renal injury (27), acute rejection (28,29), and ischemic acute kidney injury (30). MIG and IP10 were elevated significantly in hypertensive subjects, especially those with microalbuminuria (13,15,31). MIG and its receptor, CXCR3, are expressed not only in infiltrating immune cells, but also in the renal glomeruli and tubules (32,33). The stimulation of CXCR3 by MIG leads to the production of reactive oxygen species (ROS) (34). Overproduction of ROS contributes to the pathophysiology of hypertension and endothelial dysfunction, as well as renal and vascular injury (35,36). Ultimately, MIG and IP10 might be important in early renal endothelial microvascular injury in hypertensive obese children.

Consistent with our results, several cross-sectional and prospective studies have demonstrated positive associations between microalbuminuria and diastolic hypertension in overweight children (37). These findings suggest that peripheral vascular endothelial changes due to diastolic hypertension in obese hypertensive children are especially detrimental to the renal glomerular bed, resulting in increased glomerular permeability to albumin.

The study was designed to have a sufficient sample size with p α=0.05 and power of 80%, assuming the prevalence of microalbuminuria in obesity to be 2.4% in the control group and 31% among hypertensive obese cases (38,39,40). Thus, it should be necessary to study 25 obese hypertensive cases and 25 obese controls. We conducted a retrospective power calculation with power and sample size program version 3.0. The study power was calculated as 75%.

This study was limited in that we could not evaluate systemic inflammation (e.g., IL-6, IL-10, CRP, and TNF-α expression levels). Another limitation was the variability in treatment duration, because the BP control times of the patients differed. We used captopril to treat hypertension. Currently, no studies have shown a benefit of one ACE inhibitor over another for anti-hypertensive effectiveness, even in adults. However, captopril lowered not only BP, but also TG levels, LDL cholesterol levels, the left ventricular mass, and the oxidative burden level, and it improved insulin sensitivity (41,42,43,44,45).

In this study, urinary RANTES seemed to be affected by both hypertension and obesity, and urinary IP10 and MIG seemed to be affected predominantly by hypertension. Furthermore, anti-hypertensive management reversed the urinary levels of these chemokines. T cells play an important role in the pathogenesis of hypertension (7). Our study shows that Th1 cells could be activated in obese hypertensive children before the onset of clinical indicators of target-organ damage. This is the first study to evaluate urinary chemokines in hypertensive children. Additional studies on larger populations are required to confirm these preliminary results.

## Figures and Tables

**Table 1:**
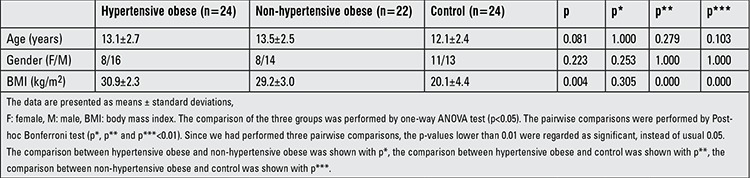
Demographic features of the study population

**Table 2:**
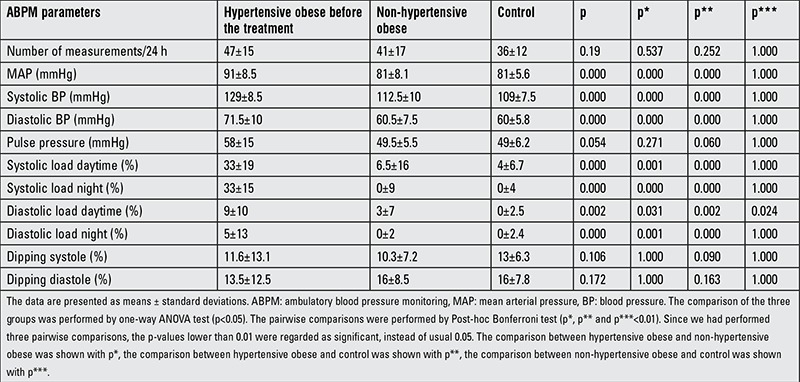
Ambulatory blood pressure monitoring parameters in the pre-treatment hypertensive obese and other groups

**Table 3:**
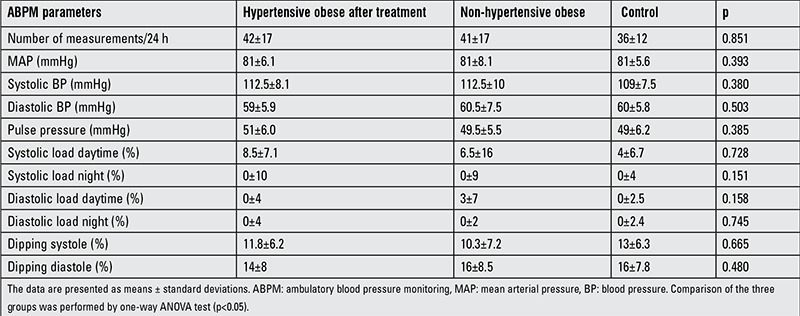
Ambulatory blood pressure monitoring parameters in the post-treatment hypertensive obese and other groups

**Table 4:**
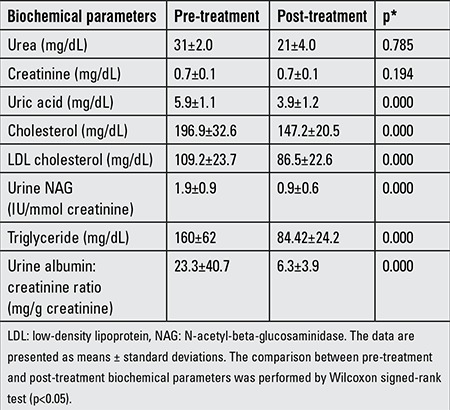
Biochemical parameters in the hypertensive obese group

**Table 5:**
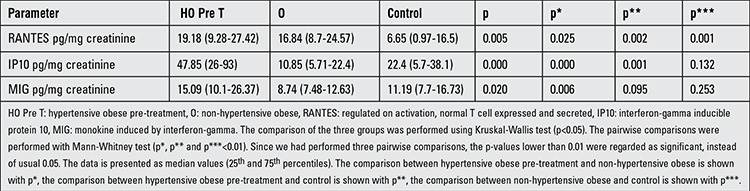
Urinary chemokine results in the pre-treatment hypertensive obese and other groups

**Table 6:**
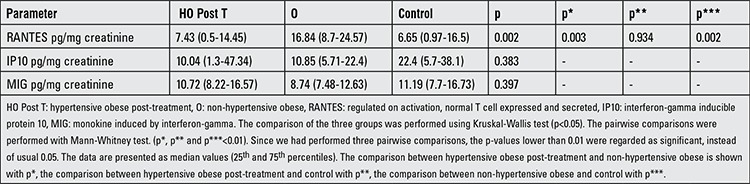
Urinary chemokine results in the post-treatment hypertensive obese and other groups
